# (1-Phenyl-1*H*-1,2,3-triazol-4-yl)methyl pyridine-3-carboxyl­ate

**DOI:** 10.1107/S1600536810021999

**Published:** 2010-06-16

**Authors:** Zakirjon Karimov, Ibrakhim Abdugafurov, Samat Talipov, Bakhodir Tashkhodjaev

**Affiliations:** a Tashkent Institute of Irrigation and Melioration, Qori-Niyoziy Str. 39, Tashkent 100000, Uzbekistan; b Andijan State University, Universitetskaja str. 129, Andijan 170100, Uzbekistan; c Institute of Bioorganic Chemistry, Academy of Sciences of Uzbekistan, Mirzo Ulugbek Str,83, Tashkent 100125, Uzbekistan; d S.Yunusov Institute of the Chemistry of Plant Substances, Academy of Sciences of Uzbekistan, Mirzo Ulugbek Str. 77, Tashkent 100170, Uzbekistan

## Abstract

In the title compound, C_15_H_12_N_4_O_2_, the dihedral angle between the planes of the nicotino­yloxy fragment and triazole ring is 88.61 (5)°. The dihedral angle between the planes of triazole and benzene rings is 16.54 (11)°. The crystal structure is stabilized by inter­molecular C—H⋯N, C—H⋯O and C—H⋯π(triazole) hydrogen bonds and aromatic π–π stacking inter­actions between the benzene and triazole rings [centroid–centroid distance = 3.895 (1) Å]

## Related literature

For the synthesis of 1,2,3-triazole derivatives, see: Berestovitskaya *et al.* (2007 ▶); Piterskaya *et al.* (1996*a* ▶,*b* ▶). For their physiological activity, see: Contreras *et al.* (1978 ▶). For related structures, see: Berestovitskaya *et al.* (2007 ▶); Monkowius *et al.* (2007 ▶). For bond-length data, see: Allen *et al.* (1987 ▶). 
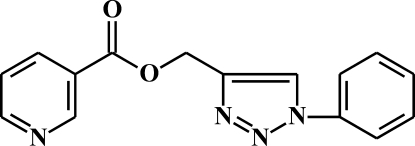


## Experimental

### 

#### Crystal data

C_15_H_12_N_4_O_2_
*M* *_r_* = 280.29
Monoclinic, 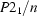

*a* = 5.5178 (5) Å
*b* = 23.650 (2) Å
*c* = 10.287 (2) Å
β = 91.841 (14)°
*V* = 1341.8 (3) Å^3^
*Z* = 4
Cu *K*α radiation
μ = 0.79 mm^−1^
*T* = 293 K
0.70 × 0.45 × 0.10 mm

#### Data collection

Oxford Diffraction Xcalibur Ruby diffractometer
Absorption correction: multi-scan (*CrysAlis PRO*; Oxford Diffraction, 2009 ▶) *T*_min_ = 0.674, *T*_max_ = 1.000
4791 measured reflections
2460 independent reflections
1877 reflections with *I* > 2σ(*I*)
*R*_int_ = 0.026

#### Refinement

*R*[*F* ^2^ > 2σ(*F* ^2^)] = 0.041
*wR*(*F* ^2^) = 0.124
*S* = 1.04
2460 reflections
191 parameters
H-atom parameters constrained
Δρ_max_ = 0.18 e Å^−3^
Δρ_min_ = −0.13 e Å^−3^

### 

Data collection: *CrysAlis PRO* (Oxford Diffraction, 2009 ▶); cell refinement: *CrysAlis PRO*; data reduction: *CrysAlis PRO*; program(s) used to solve structure: *SHELXS97* (Sheldrick, 2008 ▶); program(s) used to refine structure: *SHELXL97* (Sheldrick, 2008 ▶); molecular graphics: *XP* in *SHELXTL* (Sheldrick, 2008 ▶); software used to prepare material for publication: *SHELXL97*.

## Supplementary Material

Crystal structure: contains datablocks I, global. DOI: 10.1107/S1600536810021999/kp2262sup1.cif

Structure factors: contains datablocks I. DOI: 10.1107/S1600536810021999/kp2262Isup2.hkl

Additional supplementary materials:  crystallographic information; 3D view; checkCIF report

## Figures and Tables

**Table 1:** Hydrogen-bond geometry (Å, °) *Cg*1 is the centroid of the triazole ring.

*D*—H⋯*A*	*D*—H	H⋯*A*	*D*⋯*A*	*D*—H⋯*A*
C14—H14⋯N1^i^	0.93	2.64	3.550 (3)	165
C2—H2⋯O1^ii^	0.93	2.71	3.464 (2)	139
C15—H15⋯O1^iii^	0.93	2.68	3.559 (2)	158
C7—H7a⋯*Cg*1^iv^	0.97	2.92	3.313 (2)	106

Symmetry codes: (i) 
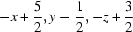
; (ii) 
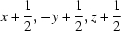
; (iii) 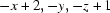; (iv) 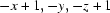.

**Fig. 1.**
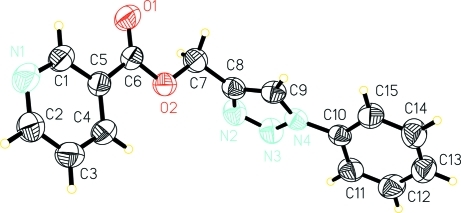
Displacement ellipsoid plot at the 50% probability level for the non-H atoms.

**Fig. 2.**
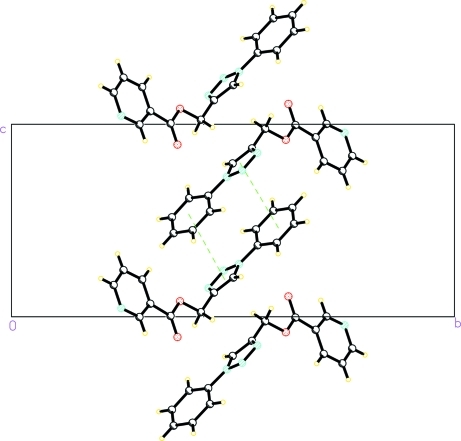
Part of the crystal structure showing a π–π stacking interactions observed between triazole and triazole rings.

## supplementary crystallographic information

### Comment

Triazole derivatives possess different biological activities (Contreras *et al.* 1978). The title compound was synthesized with purpose of finding of a potential biological active compound.

The asymmetric unit contains one molecule of 1-phenyl-4-(nicotinoyloxymethyl)-1,2,3-triazole (Fig. 1). In the molecule nicotinoyloxy, phenyl groups and triazole system are planar, with r.m.s. deviations of 0.0075, 0.0048 and 0.0028 Å, respectively. Nicotinoyloxy fragment arranged nearly perpendicular to triazole ring. The angle between the planes of the nicotinoyloxy fragment and triazole ring is 88.61 (5)°. The angle between the planes of triazole and benzene rings is 16.54 (11)°. The observed structure is stabilized by C—H···N, C—H···O and C—H···π (triazole) type hydrogen bonds (Table 1) and aromatic π–π stacking interactions. A centrosymmetric π–π stacking interactions are observed between triazole group and benzene group of molecules at *x*,*y*,*z* and 1 - *x*, -*y*, 2 - *z* where the ring-centroid separation is 3.895 (1) Å, triazole centroid distance to benzene plane is 3.429 (1) Å with ring offset of 1.847 (1) Å (Fig. 2). The bond distances and angles in molecule are in normal ranges (Allen *et al.*, 1987).

### Experimental

Mixture of 3-(nicotinoyloxy)-1-propyne (1.61 g, 0,01 mole) and fresh prepared phenylazide (1.310 g, 0,011 mole) in 30 ml toluen was heated with a backflow condenser for 6 h. Then it was cooled and precipitate were isolated by decantation. Obtained crystals were washed with ether and re-crystallized from toluen. It was obtained 78.3% yeild (2.19 g) of title compound, m.p. 96–97° C, *R*_f_ =0.53 (ether-hexane 9:1). Colorless crystals suitable for X-ray analysis were obtained from acetone by slow evaporation.

### Refinement

Carbon-bound H atoms were positioned geometrically and treated as riding on their C atoms, with C—H distances of 0.93 Å (aromatic) and 0.97 Å (CH_2_) and were refined with *U*_iso_(H) =1.2Ueq(C)]. All other non-H atoms were refined anisotropically.

### Crystal data

**Table:**

C_15_H_12_N_4_O_2_	*F*(000) = 584
*M**_r_* = 280.29	*D*_x_ = 1.388 Mg m^−^^3^
Monoclinic, *P*2_1_/*n*	Melting point: 370(2) K
Hall symbol: -P 2yn	Cu *K*α radiation, λ = 1.5418 Å
*a* = 5.5178 (5) Å	Cell parameters from 2409 reflections
*b* = 23.650 (2) Å	θ = 3.6–70.6°
*c* = 10.287 (2) Å	µ = 0.79 mm^−^^1^
β = 91.841 (14)°	*T* = 293 K
*V* = 1341.8 (3) Å^3^	Prism, colourless
*Z* = 4	0.70 × 0.45 × 0.10 mm

### Data collection

**Table:**

Oxford Diffraction Xcalibur Ruby diffractometer	2460 independent reflections
Radiation source: Enhance (Cu) X-ray Source	1877 reflections with *I* > 2σ(*I*)
graphite	*R*_int_ = 0.026
Detector resolution: 10.2576 pixels mm^-1^	θ_max_ = 70.8°, θ_min_ = 3.7°
ω scans	*h* = −6→5
Absorption correction: multi-scan (*CrysAlis PRO*; Oxford Diffraction, 2009)	*k* = −22→28
*T*_min_ = 0.674, *T*_max_ = 1.000	*l* = −12→7
4791 measured reflections	

### Refinement

**Table:**

Refinement on *F*^2^	Secondary atom site location: difference Fourier map
Least-squares matrix: full	Hydrogen site location: inferred from neighbouring sites
*R*[*F*^2^ > 2σ(*F*^2^)] = 0.041	H-atom parameters constrained
*wR*(*F*^2^) = 0.124	*w* = 1/[σ^2^(*F*_o_^2^) + (0.0774*P*)^2^ + 0.039*P*] where *P* = (*F*_o_^2^ + 2*F*_c_^2^)/3
*S* = 1.04	(Δ/σ)_max_ = 0.009
2460 reflections	Δρ_max_ = 0.18 e Å^−^^3^
191 parameters	Δρ_min_ = −0.13 e Å^−^^3^
0 restraints	Extinction correction: *SHELXL97* (Sheldrick, 2008), Fc^*^=kFc[1+0.001xFc^2^λ^3^/sin(2θ)]^-1/4^
Primary atom site location: structure-invariant direct methods	Extinction coefficient: 0.0116 (12)

### Special details

**Table:**

Experimental. CrysAlisPro, Oxford Diffraction Ltd., Version 1.171.33.40 (release 27-04-2009 CrysAlis171 .NET) (compiled Apr 27 2009,10:20:11) Empirical absorption correction using spherical harmonics, implemented in SCALE3 ABSPACK scaling algorithm.
Geometry. All e.s.d.'s (except the e.s.d. in the dihedral angle between two l.s. planes) are estimated using the full covariance matrix. The cell e.s.d.'s are taken into account individually in the estimation of e.s.d.'s in distances, angles and torsion angles; correlations between e.s.d.'s in cell parameters are only used when they are defined by crystal symmetry. An approximate (isotropic) treatment of cell e.s.d.'s is used for estimating e.s.d.'s involving l.s. planes.
Refinement. Refinement of *F*^2^ against ALL reflections. The weighted *R*-factor *wR* and goodness of fit *S* are based on *F*^2^, conventional *R*-factors *R* are based on *F*, with *F* set to zero for negative *F*^2^. The threshold expression of *F*^2^ > σ(*F*^2^) is used only for calculating *R*-factors(gt) *etc*. and is not relevant to the choice of reflections for refinement. *R*-factors based on *F*^2^ are statistically about twice as large as those based on *F*, and *R*- factors based on ALL data will be even larger.

### Fractional atomic coordinates and isotropic or equivalent isotropic displacement parameters (Å^2^)

**Table:**

	*x*	*y*	*z*	*U*_iso_*/*U*_eq_	
O1	1.0509 (3)	0.12524 (6)	0.39435 (11)	0.0714 (4)	
O2	0.8540 (2)	0.11948 (5)	0.58047 (11)	0.0523 (3)	
N1	1.4934 (3)	0.25199 (7)	0.54437 (16)	0.0742 (5)	
N2	0.3515 (3)	0.05484 (6)	0.64902 (15)	0.0623 (4)	
N3	0.2755 (2)	0.02085 (7)	0.73956 (15)	0.0594 (4)	
N4	0.4631 (2)	−0.01361 (5)	0.77328 (12)	0.0462 (3)	
C1	1.3474 (4)	0.21113 (8)	0.49855 (17)	0.0633 (5)	
H1	1.3699	0.1984	0.4143	0.076*	
C2	1.4557 (4)	0.26924 (9)	0.66483 (19)	0.0704 (5)	
H2	1.5531	0.2981	0.6987	0.084*	
C3	1.2820 (4)	0.24710 (8)	0.74223 (17)	0.0666 (5)	
H3	1.2648	0.2603	0.8265	0.080*	
C4	1.1335 (3)	0.20495 (8)	0.69328 (16)	0.0582 (5)	
H4	1.0141	0.1892	0.7437	0.070*	
C5	1.1658 (3)	0.18666 (7)	0.56780 (15)	0.0485 (4)	
C6	1.0211 (3)	0.14094 (7)	0.50420 (15)	0.0503 (4)	
C7	0.7195 (3)	0.07246 (8)	0.52359 (16)	0.0600 (5)	
H7A	0.8304	0.0464	0.4836	0.072*	
H7B	0.6066	0.0863	0.4567	0.072*	
C8	0.5854 (3)	0.04295 (7)	0.62530 (15)	0.0505 (4)	
C9	0.6565 (3)	−0.00074 (7)	0.70339 (15)	0.0498 (4)	
H9	0.8078	−0.0181	0.7075	0.060*	
C10	0.4351 (3)	−0.05578 (7)	0.87124 (15)	0.0462 (4)	
C11	0.2462 (3)	−0.05189 (8)	0.95377 (18)	0.0613 (5)	
H11	0.1349	−0.0225	0.9442	0.074*	
C12	0.2202 (3)	−0.09122 (9)	1.05070 (19)	0.0679 (5)	
H12	0.0903	−0.0888	1.1059	0.082*	
C13	0.3853 (4)	−0.13394 (9)	1.0659 (2)	0.0698 (5)	
H13	0.3697	−0.1603	1.1323	0.084*	
C14	0.5740 (4)	−0.13789 (10)	0.9831 (2)	0.0789 (6)	
H14	0.6860	−0.1671	0.9934	0.095*	
C15	0.5996 (3)	−0.09893 (9)	0.88416 (19)	0.0671 (5)	
H15	0.7266	−0.1020	0.8273	0.081*	

### Atomic displacement parameters (Å^2^)

**Table:**

	*U*^11^	*U*^22^	*U*^33^	*U*^12^	*U*^13^	*U*^23^
O1	0.0949 (10)	0.0714 (9)	0.0485 (7)	−0.0203 (7)	0.0127 (6)	−0.0026 (6)
O2	0.0533 (6)	0.0509 (7)	0.0531 (6)	−0.0076 (5)	0.0077 (5)	−0.0050 (5)
N1	0.0808 (11)	0.0768 (11)	0.0660 (10)	−0.0296 (9)	0.0194 (8)	−0.0046 (8)
N2	0.0544 (8)	0.0561 (9)	0.0766 (10)	0.0028 (7)	0.0042 (7)	0.0052 (8)
N3	0.0433 (7)	0.0591 (9)	0.0761 (10)	0.0071 (7)	0.0071 (7)	0.0038 (8)
N4	0.0375 (6)	0.0472 (7)	0.0540 (7)	0.0014 (6)	0.0038 (5)	−0.0059 (6)
C1	0.0727 (11)	0.0645 (11)	0.0532 (10)	−0.0148 (10)	0.0140 (9)	−0.0010 (8)
C2	0.0777 (13)	0.0669 (12)	0.0670 (11)	−0.0241 (10)	0.0087 (10)	−0.0064 (10)
C3	0.0748 (12)	0.0681 (12)	0.0577 (11)	−0.0128 (10)	0.0123 (9)	−0.0138 (9)
C4	0.0587 (10)	0.0604 (11)	0.0565 (10)	−0.0068 (8)	0.0168 (8)	−0.0006 (8)
C5	0.0505 (8)	0.0460 (9)	0.0493 (8)	0.0009 (7)	0.0054 (7)	0.0033 (7)
C6	0.0565 (9)	0.0480 (9)	0.0465 (9)	0.0006 (8)	0.0057 (7)	0.0053 (7)
C7	0.0680 (11)	0.0559 (10)	0.0562 (10)	−0.0140 (9)	0.0022 (8)	−0.0067 (8)
C8	0.0489 (8)	0.0488 (9)	0.0538 (9)	−0.0055 (7)	0.0013 (7)	−0.0095 (7)
C9	0.0386 (7)	0.0567 (10)	0.0543 (9)	0.0003 (7)	0.0054 (7)	−0.0045 (8)
C10	0.0418 (7)	0.0460 (8)	0.0511 (8)	−0.0025 (7)	0.0053 (6)	−0.0076 (7)
C11	0.0521 (9)	0.0629 (11)	0.0699 (11)	0.0067 (8)	0.0157 (8)	−0.0027 (9)
C12	0.0606 (10)	0.0772 (13)	0.0673 (12)	−0.0039 (10)	0.0221 (9)	−0.0022 (10)
C13	0.0723 (12)	0.0686 (13)	0.0693 (12)	−0.0005 (10)	0.0130 (10)	0.0088 (10)
C14	0.0784 (13)	0.0700 (13)	0.0899 (15)	0.0212 (11)	0.0255 (11)	0.0180 (11)
C15	0.0612 (10)	0.0679 (12)	0.0738 (12)	0.0135 (10)	0.0261 (9)	0.0072 (10)

### Geometric parameters (Å, °)

**Table:**

O1—C6	1.2059 (19)	C5—C6	1.484 (2)
O2—C6	1.3301 (19)	C7—C8	1.476 (2)
O2—C7	1.450 (2)	C7—H7A	0.9700
N1—C2	1.327 (2)	C7—H7B	0.9700
N1—C1	1.334 (2)	C8—C9	1.359 (2)
N2—N3	1.309 (2)	C9—H9	0.9300
N2—C8	1.351 (2)	C10—C11	1.369 (2)
N3—N4	1.3536 (18)	C10—C15	1.369 (2)
N4—C9	1.3409 (19)	C11—C12	1.375 (3)
N4—C10	1.430 (2)	C11—H11	0.9300
C1—C5	1.376 (2)	C12—C13	1.366 (3)
C1—H1	0.9300	C12—H12	0.9300
C2—C3	1.369 (3)	C13—C14	1.369 (3)
C2—H2	0.9300	C13—H13	0.9300
C3—C4	1.375 (2)	C14—C15	1.383 (3)
C3—H3	0.9300	C14—H14	0.9300
C4—C5	1.378 (2)	C15—H15	0.9300
C4—H4	0.9300		
			
C6—O2—C7	114.22 (13)	O2—C7—H7B	109.7
C2—N1—C1	116.19 (16)	C8—C7—H7B	109.7
N3—N2—C8	109.28 (14)	H7A—C7—H7B	108.2
N2—N3—N4	107.01 (13)	N2—C8—C9	108.11 (15)
C9—N4—N3	109.94 (14)	N2—C8—C7	122.22 (16)
C9—N4—C10	129.94 (13)	C9—C8—C7	129.55 (16)
N3—N4—C10	120.13 (13)	N4—C9—C8	105.66 (14)
N1—C1—C5	124.29 (17)	N4—C9—H9	127.2
N1—C1—H1	117.9	C8—C9—H9	127.2
C5—C1—H1	117.9	C11—C10—C15	120.38 (17)
N1—C2—C3	123.98 (18)	C11—C10—N4	119.46 (15)
N1—C2—H2	118.0	C15—C10—N4	120.15 (15)
C3—C2—H2	118.0	C10—C11—C12	120.25 (17)
C2—C3—C4	118.94 (17)	C10—C11—H11	119.9
C2—C3—H3	120.5	C12—C11—H11	119.9
C4—C3—H3	120.5	C13—C12—C11	119.91 (18)
C3—C4—C5	118.49 (16)	C13—C12—H12	120.0
C3—C4—H4	120.8	C11—C12—H12	120.0
C5—C4—H4	120.8	C12—C13—C14	119.8 (2)
C1—C5—C4	118.09 (16)	C12—C13—H13	120.1
C1—C5—C6	117.97 (15)	C14—C13—H13	120.1
C4—C5—C6	123.91 (15)	C13—C14—C15	120.7 (2)
O1—C6—O2	123.60 (16)	C13—C14—H14	119.7
O1—C6—C5	123.36 (16)	C15—C14—H14	119.7
O2—C6—C5	113.04 (14)	C10—C15—C14	119.00 (17)
O2—C7—C8	109.78 (13)	C10—C15—H15	120.5
O2—C7—H7A	109.7	C14—C15—H15	120.5
C8—C7—H7A	109.7		
			
C8—N2—N3—N4	−0.57 (18)	N3—N2—C8—C7	177.18 (14)
N2—N3—N4—C9	0.18 (18)	O2—C7—C8—N2	94.82 (18)
N2—N3—N4—C10	179.94 (13)	O2—C7—C8—C9	−89.6 (2)
C2—N1—C1—C5	−0.2 (3)	N3—N4—C9—C8	0.28 (18)
C1—N1—C2—C3	1.1 (3)	C10—N4—C9—C8	−179.45 (15)
N1—C2—C3—C4	−1.0 (3)	N2—C8—C9—N4	−0.62 (17)
C2—C3—C4—C5	0.0 (3)	C7—C8—C9—N4	−176.70 (15)
N1—C1—C5—C4	−0.8 (3)	C9—N4—C10—C11	162.57 (16)
N1—C1—C5—C6	−178.98 (18)	N3—N4—C10—C11	−17.1 (2)
C3—C4—C5—C1	0.8 (3)	C9—N4—C10—C15	−15.9 (3)
C3—C4—C5—C6	178.92 (16)	N3—N4—C10—C15	164.39 (16)
C7—O2—C6—O1	4.0 (2)	C15—C10—C11—C12	0.2 (3)
C7—O2—C6—C5	−176.54 (14)	N4—C10—C11—C12	−178.28 (16)
C1—C5—C6—O1	−1.8 (3)	C10—C11—C12—C13	0.9 (3)
C4—C5—C6—O1	−179.94 (17)	C11—C12—C13—C14	−1.0 (3)
C1—C5—C6—O2	178.69 (15)	C12—C13—C14—C15	0.2 (3)
C4—C5—C6—O2	0.6 (2)	C11—C10—C15—C14	−1.1 (3)
C6—O2—C7—C8	165.90 (14)	N4—C10—C15—C14	177.40 (17)
N3—N2—C8—C9	0.76 (18)	C13—C14—C15—C10	0.9 (3)

### Hydrogen-bond geometry (Å, °)

**Table:**

Cg1 is the centroid of the triazole ring.

**Table:**

*D*—H···*A*	*D*—H	H···*A*	*D*···*A*	*D*—H···*A*
C14—H14···N1^i^	0.93	2.64	3.550 (3)	165
C2—H2···O1^ii^	0.93	2.71	3.464 (2)	139
C15—H15···O1^iii^	0.93	2.68	3.559 (2)	158
C7—H7a···Cg1^iv^	0.97	2.917	3.313 (2)	106

Symmetry codes: (i) −*x*+5/2, *y*−1/2, −*z*+3/2; (ii) *x*+1/2, −*y*+1/2, *z*+1/2; (iii) −*x*+2, −*y*, −*z*+1; (iv) −*x*+1, −*y*, −*z*+1.
